# Concurrent Indicators of Gait Velocity and Variability Are Associated with 25-Year Cognitive Change: A Retrospective Longitudinal Investigation

**DOI:** 10.3389/fnagi.2017.00017

**Published:** 2017-02-13

**Authors:** Stuart W. S. MacDonald, Sandra Hundza, Janet A. Love, Correne A. DeCarlo, Drew W. R. Halliday, Paul W. H. Brewster, Timothy V. Lukyn, Richard Camicioli, Roger A. Dixon

**Affiliations:** ^1^Department of Psychology, University of VictoriaVictoria, BC, Canada; ^2^Institute of Aging and Lifelong Health, University of VictoriaVictoria, BC, Canada; ^3^School of Exercise Science, University of VictoriaVictoria, BC, Canada; ^4^Department of Medicine (Neurology), University of AlbertaEdmonton, AB, Canada; ^5^Neuroscience and Mental Health Institute, University of AlbertaEdmonton, AB, Canada; ^6^Department of Psychology, University of AlbertaEdmonton, AB, Canada

**Keywords:** cognitive change, physical function, gait, variability, Victoria Longitudinal Study

## Abstract

**Background/Objectives**: Physical function indicators, including gait velocity, stride time and step length, are linked to neural and cognitive function, morbidity and mortality. Whereas cross-sectional associations are well documented, far less is known about long-term patterns of cognitive change as related to objective indicators of mobility-related physical function.

**Methods**: Using data from the Victoria Longitudinal Study, a long-term investigation of biological and health aspects of aging and cognition, we examined three aspects of cognition-physical function linkages in 121 older adults. First, we examined a simple marker of physical function (3 m timed-walk) as a predictor of cross-sectional differences and up to 25-year change for four indicators of cognitive function. Second, we tested associations between two markers of gait function derived from the GAITRite system (velocity and stride-time variability) and differences and change in cognition. Finally, we evaluated how increasing cognitive load during GAITRite assessment influenced the associations between gait and cognition.

**Results**: The simple timed-walk measure, commonly used in clinical and research settings, was a minor predictor of change in cognitive function. In contrast, the objectively measured indicator of walking speed significantly moderated long-term cognitive change. Under increasing cognitive load, the moderating influence of velocity on cognitive change increased, with increasing variability in stride time also emerging as a predictor of age-related cognitive decline.

**Conclusion**: These findings: (a) underscore the utility of gait as a proxy for biological vitality and for indexing long-term cognitive change; and (b) inform potential mechanisms underlying age-related linkages in physical and cognitive function.

## Introduction

Differences and change in cognition have recently been explained by *functional biomarkers*—select biological processes that systematically decline with age, index functional capacity of various bodily systems and can be objectively measured (Anstey, 2008; Dixon, 2011; MacDonald et al., 2011). Recently, temporal and spatial measures of gait have shown promise for informing patterns and mechanisms of cognitive aging. Measures of gait speed, assessed in various ways (timed 3 m walk, computerized walkway), have been linked to cognitive and functional impairment (Rosano et al., 2008), health (Abellan Van Kan et al., 2009), and mortality risk (Studenski et al., 2011). Among the reasons for observed links between gait and deleterious age-related outcomes, individual differences in gait may reflect the integrity of underlying organ systems including respiratory, vascular, nervous, circulatory and musculoskeletal (Abellan Van Kan et al., 2009; Studenski et al., 2011). Beyond the relative health of individual organ systems, walking also places demands on energy metabolism, with links between diminished gait speed and various age-related outcomes due to higher metabolic costs for the elderly (Mian et al., 2006). Further, the central and peripheral nervous systems share associations with gait function, with diminished gait velocity or increased variability linked to CNS impairments (dementia, Parkinson’s disease); subclinical infarcts and white matter abnormalities have also been linked to indicators of gait function (Rosano et al., 2007). Ultimately, a better understanding of how changes in various bodily systems and the CNS influence mobility constraints may also inform our understanding of age-CNS-cognition associations (Rosso et al., 2013).

Cross-sectional studies have linked slowing gait velocity to poorer cognitive function in elderly samples (Martin et al., 2013), as well as to age-related pathological outcomes including mild cognitive impairment (MCI) and dementia (Hausdorff and Buchman, 2013). Cognitive processes including executive function, attention, and processing speed share the strongest associations with gait, with observed gait-cognition associations perhaps reflecting a common neural substrate—mediation by frontal brain circuits sensitive to aging pathologies including vascular and neurodegenerative diseases (Parihar et al., 2013). Similarly, increasing gait variability (within-person fluctuations in gait characteristics across steps on a computerized walkway) has been linked to lower levels of physical activity and mobility for older adults (Brach et al., 2010), increased risk of falling (Callisaya et al., 2011), and ultimately to cognitive impairment (Hausdorff and Buchman, 2013). In fact, gait disturbances and falls often serve as an *index event* for facilitating early detection of cognitive impairment or diagnosing dementia (Axer et al., 2010). Finally, evidence from dual-task studies underscores the important association between gait and cognitive function (Killane et al., 2014). Requiring participants to engage in a cognitive task (counting backwards by 7 s from 100) while simultaneously walking negatively impacts both gait and cognition (Yogev-Seligmann et al., 2008). This dual-task cost increases with age, perhaps reflecting concerns that older adults have about falls. Such concerns may lead to increased focus on the act of walking itself, requiring attentional processes and top-down cognitive control that impairs the self-organizing dynamics of the motor system (Lövdén et al., 2008). With increasing age, sensorimotor functions including gait require an increasing number of cognitive resources. Thus, increasing cognitive task difficulty may result in poorer motor function due to cross-domain resource competition, particularly for older adults with diminished cognitive resources (Schaefer et al., 2006).

Despite the burgeoning research interest in gait-related physical function and cognition in aging, the associations across long-term change periods remain poorly understood (Mielke et al., 2013). Recent evidence suggests that slowing gait speed precedes cognitive declines during the prodromal phase of dementia (Hausdorff and Buchman, 2013). However, few studies have examined longitudinal gait-cognition associations, particularly for community-dwelling elderly (Clouston et al., 2013). Using multi-wave data from the Victoria Longitudinal Study (VLS), we examined gait-cognition linkages guided by three research objectives. The first research objective tested whether individual differences for a simple measure of gait speed, time required to walk a distance of 3 m, predicts age differences and change in cognitive function spanning numerous waves of assessment. Extending previous cross-sectional research (Clouston et al., 2013), the second objective assessed individual differences for GAITRite-derived measures (normalized velocity, stride-time variability) as predictors of age differences and change in cognitive function. A related question addressed whether cognitive function was similarly predicted by the simple timed walk vs. the GAITRite derived measure of walking speed. The expectation is that the GAITRite system will yield a more precise measurement for use in research contexts. The third research objective tested the moderating impact of increasing cognitive load on gait performance (walk-only condition vs. 7-letter words spelled backwards) and its corresponding association with differences and change in cognitive function. The overloading of the CNS by additional task demands may not only influence the impact on various gait indicators, but gait-cognition associations as well (Rosano et al., 2007; Lövdén et al., 2008). Dual-task studies examining how cognitive performance is further influenced by walking and performing a task at the same time have demonstrated that higher cognitive load may hamper motor control due to cross-domain resource competition (Schaefer et al., 2006). We expected that such resource competition would also increase the predictive association of gait on long-term cognitive change.

## Materials and Methods

### Participants

This study uses data from the VLS, a long-term project examining biological, health and neurocognitive aspects of aging. All data collection procedures are in full compliance with prevailing institutional research board ethic guidelines. At intake, VLS participants are community-dwelling adults, aged 55–85 years, with no serious health conditions (baseline exclusionary criteria include dementia diagnosis, as well as serious cardiovascular or cerebrovascular conditions). The present study was based upon data from VLS Samples 1 (initiated in 1986) and 2 (initiated in 1992), as it provided the largest number of retest waves and the overall longest duration of archival data. The research design of the VLS calls for retest intervals of approximately 3 years, with the present study sample spanning up to eight waves and as many as 25-years of longitudinal follow-up. The GAITRite^®^ computerized walkway was first administered in the VLS protocol for Sample 1 Wave 8 (S1W8) and Sample 2 Wave 6 (S2W6), thereby facilitating the investigation of concurrent gait function with retrospective cognitive change.

Across the combined samples, gait assessment was completed by 121 participants (78 women, 43 men) aged 75–97 years (*M*_age_ = 84.92 years; SD = 4.74). Despite the advanced age of the S1W8 and S2W6 returnees, this group exhibited select profiles of education (*M* = 14.99 years, SD = 3.04) and health (self-reported health relative to perfect, *M* = 0.98, SD = 0.83; and relative to same-aged peers, *M* = 0.69, SD = 0.76) on a 5-point scale (ranging from 0 = very good to 4 = very poor). Education and health background characteristics for the original S1W1 (*n* = 484) and S2W1 (*n* = 530) samples, respectively, were comparable: years of education (*M* = 13.42, SD = 3.09; *M* = 14.81, SD = 3.15), self-reported health relative to perfect (*M* = 0.83, SD = 0.76; *M* = 0.77, SD = 0.73), and self-reported health relative to same-aged peers (*M* = 0.63, SD = 0.71l, *M* = 0.58, SD = 0.70). Using analysis of variance, we contrasted group differences in the baseline education and health measures for the current sample (those who returned for all waves of testing; *n* = 127) vs. those who attrited at any point during the study after baseline assessment (*n* = 887). As expected, at baseline, the final-sample returnees had more years of education (*M* = 14.88 vs. 14.05, *F*_(1,1012)_ = 7.47, *p* < 0.01) and reported themselves to be in better health relative to perfect (*M* = 0.63 vs. 0.82, *F*_(1,1012)_ = 7.44, *p* < 0.01), but not relative to their peers (n.s.). These group differences notwithstanding, very high levels of education and self-reported health (very good to good range) were reported for both the returnees as well as those who dropped out of the study.

### Measures

#### Cognitive Function

Participants completed measures of perceptual speed (Digit Symbol Substitution), episodic memory (word recall), incidental memory (incidental recall of the digit symbol coding key), and semantic memory (vocabulary). Assessments of episodic and semantic memory were available across all waves for both samples, with up to 6 waves of data available for perceptual speed and incidental memory for Sample 1 (W3–W8) and Sample 2 (W1–W5).

##### Perceptual speed

The Digit Symbol Substitution test from the Wechsler Adult Intelligence Scale (Wechsler, 1958) was administered to index perceptual processing speed. Participants were given 90 s to transcribe as many symbols as possible into the empty boxes based on the digit–symbol associations specified in the coding key.

##### Episodic memory

The word recall test was based on six categorized lists of common English nouns from established norms (Howard, 1980). Each word list consisted of six words from five taxonomic categories typed on a single page in unblocked order. Participants were given 2 min to study each list and 5 min for free recall.

##### Incidental memory

Following completion of the digit symbol test, participants were presented with a coding key containing only the nine unique symbols. The incidental test of memory required participants to recall the number corresponding to each of the nine unique symbols based on the associations specified in the original coding key. Participants were given 90 s for recall.

##### Semantic memory

English vocabulary was indexed by a 54-item recognition measure adapted from the Kit of Factor Referenced Cognitive Tests (Ekstrom et al., 1976). Participants were given 15 min to complete the test.

### Gait and Mobility

#### Timed Walk

A basic measure of walking speed was assessed reflecting the time (in seconds) required to walk a distance of 3 m, recorded using a handheld stopwatch. Participants began walking from a stationary position behind a clearly demarcated line, and proceeded in one direction until they walked beyond a second line. Participants did not decelerate at the 3 m marker, as ample space (>1.5 m) was available beyond this point.

#### GAITRite Computerized Walkway

The indicators of gait speed and variability were derived from a 4.88 m GAITRite^®^ instrumented walkway (GAITRite; CIR Systems, Sparta, NJ, USA). Each sensor pad has an active area of 60 cm square and contains 2304 sensors arranged in a 121.9 × 121.9 cm grid pattern. Sensors are activated under pressure at footfall and deactivated at toe-off, enabling capture of the relative arrangement of footfalls as a function of time. Gait data from the pressure-activated sensors were sampled at 120 Hz and transferred to a computer for subsequent processing using GAITRite Platinum software (CIR Systems Inc, 2010).

Participants walked at their normal pace on the instrumented walkway, while wearing their own comfortable shoes, in a well-lit environment. No practice passes on the gait walkway were allowed prior to commencing testing. Participants completed two complete back-and-forth circuits (four total passes) of the mat for each condition; walking commenced 1.5 m prior to the mat and concluded 1.5 m beyond the mat to permit sufficient time for acceleration and deceleration. Data from the four passes for each cognitive condition were concatenated.

##### Gait speed

Normalized velocity from the GAITRite walkway was computed by dividing total distance traveled by the ambulation time (indexed in centimeters per second), and by then standardizing this estimate through division by the average leg length (yielding a normalized estimate in units of leg length per second) for each participant to control for individual differences in height.

##### Gait speed variability

This variability estimate from the GAITRite walkway was calculated on the basis of stride time, reflecting the time elapsed (in seconds) between the initial contacts of two consecutive footfalls of the same foot. Intraindividual variability in stride time was computed as the coefficient of variation (CV: the within-person standard deviation divided by the within-person mean) to control for mean differences as a potential confound.

##### Cognitive load condition

Participants completed two separate walking conditions on the GAITRite walkway: a walk-only (no load) condition, as well as a walk condition performed while simultaneously completing a cognitive task (load condition). For each condition, participants completed two complete back-and-forth circuits (four total passes) of the mat. The cognitive task in the load condition required participants to spell 7-letter words backwards that were equated for a grade 7 to 9 reading level. The no load condition always preceded the load condition.

### Statistical Analyses

We used HLM 6.08 software (Scientific Software International, 2004) to fit linear mixed models to test the research objectives. To examine whether each of the cognitive constructs exhibited significant longitudinal changes, within-person (Level 1) models were fit for linear change as a function of time (years) in study (see equation 1). Cognitive performance for a given individual (i) at a given time (j) was modeled as a function of that individual’s performance at baseline testing (intercept centered at a representative value in the sample for Wave 1), plus his/her average individual rate of change per each additional year in the study (the slope), plus an error term.

For measures exhibiting significant change, we further examined how cognitive change spanning the up to 25-year period was related to concurrent markers of physical function (see Level 2 predictors for equation 1) including timed walk (research objective one) as well as markers of gait speed and variability (research objective two). To estimate

(1) $$\text{Level 1: }{\text{Cognition}}_{\text{ij}}\;=\;\beta_{\text{0i}}\;+\;\beta_{\text{li}}\;(\text{Time in }{\text{Study}}_{\text{ij}})\;+\;{\text{e}}_{\text{ij}}$$

$$Level\;2\;:\;\beta_{\text{0i}}\;=\;\gamma_{\text{00}}\;+\;\gamma_{\text{01}}\;(\text{Gait})\;+\;\gamma_{\text{02}}\;(\text{Age})\;+\;{\text{u}}_{\text{0i}}$$

$$\beta_{\text{1i}}\;=\;\gamma_{\text{10}}\;+\;\gamma_{\text{11}}\;(\text{Gait})\;+\;\gamma_{\text{12}}(\text{Age})\;+\;{\text{u}}_{\text{1i}}$$

average effects for the entire sample, the Level 1 individual growth parameters for intercept (*β*_0i_) and slope (*β*_1i_) were employed as to-be-predicted outcomes for the Level 2 between-person equations. Our focus concerned whether individual differences for various gait indicators were associated with cross-sectional (intercept) differences in cognitive performance or with individual differences in trajectories of cognitive change (slope). For the level-2 analysis for intercept, a given individual’s predicted cognitive performance for each measure (B_0i_) was modeled as a function of cognitive performance at centered grand-mean values for age at time of gait testing and gait (γ_00_), the average difference in performance for a 1 unit increase in gait (γ_01_) and age (γ_02_), plus a random effect (U_0i_) that estimates the variability about that sample mean holding age and gait constant. Similarly, for the level-2 analysis for slope, we modeled the predicted linear rate of change in cognitive performance for each individual (B_1i_) as a function of the average cognitive change for centered values of gait and age (γ_10_), the average difference in cognitive change per unit increase in gait (γ_11_) and age (γ_12_), plus a random effect term (U_1i_) reflecting variance about cognitive performance slopes independent of other predictors. For all level 2 models, chronological age at time of gait testing was entered as a covariate to adjust for between-person age effects. Parameters were estimated using full information maximum likelihood.

## Results

**Figure 1:**
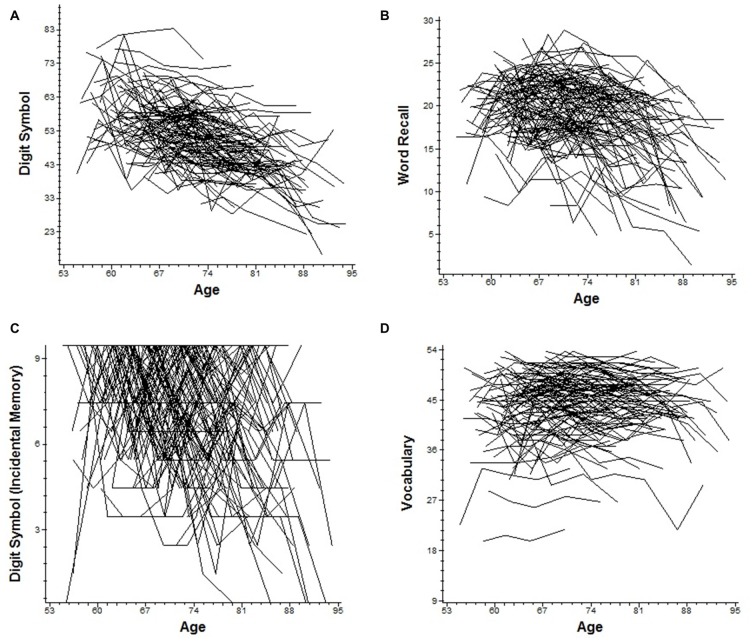
**Trajectories of age-related cognitive change over 25 years.** Individual raw slopes are plotted for all possible cases. **(A)** Digit symbol substitution: number correct. **(B)** Word recall. **(C)** Digit symbol substitution: incidental recall. **(D)** Vocabulary.

We fit linear mixed models to document up to 25-year change separately for each of the four cognitive indicators. Significant age-related declines were observed for all cognitive outcomes under consideration (see Figure 1). Each additional year in the study was associated with significant cognitive declines in perceptual speed, episodic memory, incidental recall and semantic memory (see Table 1). Between-person differences in age at time of gait testing significantly moderated intercepts for digit symbol (increasing age linked to fewer correct responses) and vocabulary (increasing age associated with more words recognized); between-person age differences also moderated two slope terms, with each additional year older associated with fewer vocabulary terms and words recalled (*p* < 0.01).

**Table 1:** **Change in cognitive performance spanning as many as 25 years of assessment**.

Variables	Intercept *γ*_00_	Slope *γ*_10_	SE Slope	*p*
Digit symbol: correct	55.48	−0.510	0.038	<0.001
Word recall	20.23	−0.167	0.019	<0.001
Digit symbol: Inc. recall	7.44	−0.065	0.013	<0.001
Vocabulary	45.22	−0.083	0.015	<0.001

*γ_00_ = Average cognitive performance centered at baseline testing and the grand mean of age; γ_10_ = average rate of linear change per additional year in study; SE = robust standard errors. Cognitive coefficients reflect words recalled (maximum score of 30), correctly paired digits to symbols (total correct in 90 s), and correct items on the vocabulary test (maximum score of 54). All intercept values were significantly different from 0, as were corresponding variance components for intercept (*p* < 0.001) and slope (*p* < 0.05). Age at time of gait testing, centered at the grand mean (84.92 years; SD = 4.74), was entered as a covariate for all models to adjust for between person age effects. Inc. = incidental recall of digit symbol coding key*.

To address our first research objective, we examined the basic, concurrently assessed (S1W8 and S2W6) timed-walk indicator as a between-person predictor of differences and change in cognitive function. For modeling purposes, concurrent timed walk was centered at the grand mean (*M* = 9.29 s; SD = 3.27). This simple measure of timed walk was not significantly associated with individual differences in cognitive function for any of the outcome measures (all two-tailed *p*’s > 0.10 for intercepts) independent of age at testing. Significant moderating effects were observed for up to 25-year change in two cognitive outcomes; each additional second increase (slowing) in timed walk above the grand mean was associated with a further 0.037 (SE = 0.013, *p* < 0.01) unit decline in digit symbol performance accuracy, as well as a further 0.011 (SE = 0.006, *p* < 0.05) unit decline in number of words successfully recalled.

**Figure 2:**
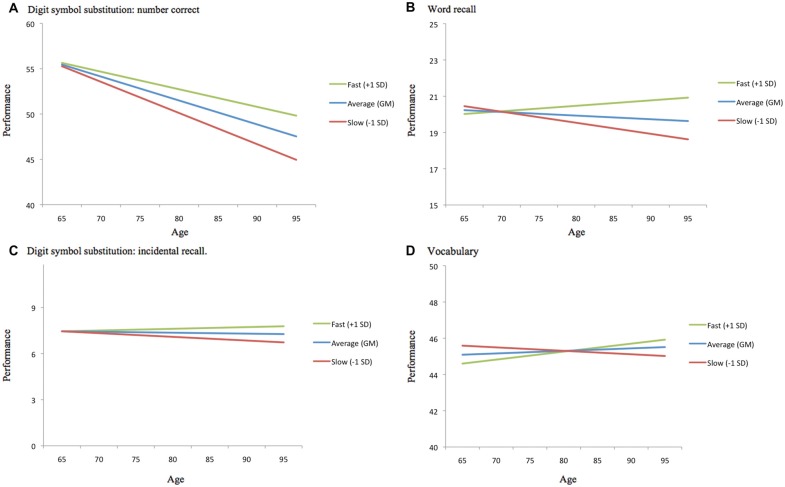
**Moderating effect of normalized gait velocity on age-related cognitive change.** For each cognitive outcome, age-related change is plotted as a function of *average* (grand mean at wave 8), *slower than average* (1 SD slower than the grand mean at wave 8), and *faster than average* (1 SD faster than the grand mean at wave 8) normalized gait velocity. **(A)** Digit symbol substitution: number correct. **(B)** Word recall. **(C)** Digit symbol substitution: incidental recall. **(D)** Vocabulary.

Our second research objective examined the two GAITRite indices as predictors of individual differences and multi-year change in cognitive function for the walk-only (no load) condition. Differences in concurrent markers of gait speed and variability did not moderate cross-sectional differences in cognitive function; there were no significant effects of gait on intercept estimates for cognitive performance, controlling for individual differences in age. However, individual differences in the GAITRite assessment of gait velocity and stride time variability significantly moderated age-related change in cognitive function (see Table 2). Per additional year in the study, a slower normalized gait velocity was associated with faster cognitive decline for word recall, digit symbol accuracy (one-tailed p-value), and vocabulary, with increased stride time variability linked to faster cognitive decline for vocabulary. The moderating influence of normalized velocity on age-related cognitive change are plotted in Figure 2 for select values (simple effects) of gait speed. Across cognitive outcomes, the significant interactions (see γ_11_ slopes in Table 2) indicate that cognitive declines across time are more pronounced for slower gait velocities.

**Table 2:** **Change in cognitive performance for the no load condition as a function of gait speed and variability**.

Variables	Intercept *γ*_00_	Slope *γ*_10_ *γ*_11_	SE	*p*
**Digit symbol: correct**
Normalized velocity	55.52	−0.510	0.038	<0.001
		0.244	0.141	<0.05*
Stride time variability	55.51	−0.508	0.039	<0.001
		−0.122	0.225	n.s.
**Word recall**
Normalized velocity	20.25	−0.165	0.018	<0.001
		0.151	0.050	<0.01
Stride time variability	20.24	−0.163	0.019	<0.001
		−0.108	0.072	n.s.
**Digit symbol: Inc. recall**
Normalized velocity	7.43	−0.064	0.012	<0.001
		0.059	0.043	n.s.
Stride time variability	7.43	−0.064	0.012	<0.001
		0.191	0.132	n.s.
**Vocabulary**
Normalized velocity	45.21	−0.083	0.015	<0.001
		0.103	0.038	<0.01
Stride time variability	45.20	−0.082	0.015	<0.001
		−0.114	0.046	<0.05

γ_00_ = Average cognitive performance centered at baseline testing for the grand mean of a given gait predictor; γ_10_ = time in study slope (average rate of linear change per additional year in study, independent of a given gait marker’s influence and age at testing); γ_11_ = time in study *gait slope interaction (estimated change in cognitive function per unit change in a given gait indicator); SE = robust standard errors. All intercept values were significantly different from 0, as were corresponding variance components. Age at time of gait testing, centered at the grand mean (84.92 years; SD = 4.74), was entered as a covariate for all models to adjust for between person age effects. Inc. = incidental recall of digit symbol coding key. *p < 0.05, one-tailed.

For our third research objective, we examined the impact of increasing cognitive load on gait-cognition associations. We replicated all aforementioned analyses conducted for the no load (walk-only) condition, and then compared key parameters of interest to assess the impact of adding cognitive load while walking on the GAITRite walkway. Similar to the no load condition findings, cross-sectional estimates of cognitive function were not associated with concurrent gait velocity; a 1 SD increase in stride-time variability was significantly related to lower intercept values in cognitive performance for word recall (2.21 fewer words recalled, *p* < 0.05) and vocabulary (4.01 fewer words recognized, *p* < 0.05). Even under increased cognitive load, the impact of gait function on individual differences in cognitive performance was modest. In contrast, gait uniformly and significantly moderated cognitive change (see Table 3). Consistent with the no-load effects of gait on change slopes (γ_11_) reported in Table 2, a slower gait velocity (relative to the sample average walking speed) was associated with accelerated cognitive decline for all four cognitive measures. Moreover, the magnitude of these slope estimates for the time × gait velocity interaction (γ_11_ estimates in Table 3) showed uniform increases under cognitive load: word recall (from 0.151 to 0.190), digit symbol correct (from 0.244 to 0.399), digit symbol incidental recall (from 0.059 to 0.109), and vocabulary (from 0.103 to 0.146).

**Table 3:** **Change in cognitive performance for the load condition as a function of select markers of gait speed and variability**.

Variables	Intercept *γ*_00_	Slope *γ*_10_ *γ*_11_	SE	*p*
**Digit symbol: correct**
Normalized velocity	55.51	−0.512	0.037	<0.001
		0.399	0.166	<0.05
Stride time variability	55.48	−0.509	0.037	<0.001
		−0.342	0.079	<0.001
**Word recall**
Normalized velocity	20.24	−0.164	0.017	<0.001
		0.190	0.055	<0.01
Stride time variability	20.23	−0.163	0.018	<0.001
		−0.104	0.038	<0.01
**Digit symbol: Inc. recall**
Normalized velocity	7.45	−0.064	0.012	<0.001
		0.109	0.047	<0.05
Stride time variability	7.45	−0.064	0.012	<0.001
		−0.093	0.028	<0.01
**Vocabulary**
Normalized velocity	45.07	−0.085	0.014	<0.001
		0.146	0.047	<0.01
Stride time variability	45.06	−0.085	0.014	<0.001
		−0.120	0.033	<0.01

γ_00_ = Average cognitive performance centered at baseline testing for the grand mean of a given gait predictor; γ_10_ = time in study slope (average rate of linear change per additional year in study, independent of a given gait marker’s influence and age at testing); γ_11_ = time in study *gait slope interaction (estimated change in cognitive function per unit change in a given gait indicator); SE = robust standard errors. All intercept values were significantly different from 0, as were corresponding variance components. Age at time of gait testing, centered at the grand mean (84.92 years; SD = 4.74), was entered as a covariate for all models to adjust for between person age effects. Inc. = incidental recall of digit symbol coding key.

Finally, in contrast to the single significant effect observed for the no load condition, under cognitive load increasing stride-time variability was consistently associated with further increases in cognitive decline (see Table 3). Each unit increase in gait variability above the sample average was associated with increased decline for word recall (64% increase in decline relative to the sample average), digit symbol correct (67% increase), digit symbol incidental recall (145% increase), and even for the knowledge-based vocabulary measure (141% increase).

## Discussion

Across the up to eight waves and 25-year follow up, significant age-related decline in cognitive function was observed, despite the select survival of the sample. Having demonstrated significant age-related cognitive decline and variance in change, gait-related physical function predictors of this change were evaluated. The first research objective tested the basic 3 m timed-walk variable as a predictor of age-related differences and change in cognitive function. Slower walking speed on the basic timed-walk variable shared a modest association with two indicators of cognitive decline (digit symbol accuracy and word recall).

In the second objective, we further tested select markers of gait velocity and variability from the GAITRite system as predictors of differences and change in cognition, and directly compared predictive patterns from the GAITRite system to the simple timed-walk task. GAITRite indicators of diminished gait velocity and increased gait variability were linked to prior 25-year accelerated cognitive decline. Among the implications, the findings are consistent with claims that select gait indicators provide a snapshot of the integrity of various bodily systems (6), and that gait may prove useful as a proxy for biological vitality and its associated underpinnings for successful cognitive aging (2, 3). Further, we extended findings by Youdas et al. (2006) by directly comparing results from the simple timed measure of gait to those from the GAITRite system. Relative to the pattern of moderating effects observed for the simple timed-walk measure, the GAITRite index of normalized velocity from the no load condition yielded significant effects for the same cognitive outcomes, as well as significantly moderated cognitive change for one additional cognitive outcome (vocabulary). Consistent with conclusions drawn by Youdas et al. (2006), these patterns support claims that data from computerized walkways provide more nuanced, precise, and reliable assessment of gait that may in turn improve sensitivity for detecting various age-related or clinical outcomes (cognitive impairment, dementia, falls, death).

A third objective of the present study was to assess gait under two distinct walking conditions (a walk-only condition vs. a condition that required simultaneous performance of a cognitive task), and to test how such increases in cognitive load during GAITRite assessment influenced associations between gait and cognitive change. Increasing cognitive load impacted gait-cognition associations in several ways. First, increasing load exerted an additional negative impact on gait-cognition associations, consistent with patterns reported for dual-task research designs (Toulotte et al., 2006). Slower gait velocity was linked to cognitive decline in the no-load condition for three of four cognitive outcomes, with the magnitude of these declines amplified under increasing cognitive load. Further, whereas stride-time variability predicted decline for a single cognitive outcome (vocabulary) in the no-load condition, significant associations were observed for all cognitive outcomes under increased load. With regard to predicting a cognitive decline, the pattern of increased associations for gait speed and emerging importance of gait variability under increased cognitive load is consistent with explanations based on resource competition (Schaefer et al., 2006). Specifically, dual-task studies have examined how performance is influenced by walking and performing a cognitive task simultaneously. Among the explanations, increased cognitive load may hamper basic motor control due to cross-domain (physical function vs. cognitive) resource competition (Woollacott and Shumway-Cook, 2002; Schaefer et al., 2006). Attentional control is of central importance for gait and the rhythmic stepping mechanism (Lindenberger et al., 2000; Woollacott and Shumway-Cook, 2002; Dubost et al., 2006). With increasing age, available cognitive resources (particularly executive processes and attentional control) are declining, with any further demands placed on these limited resources likely to negatively impact gait performance (Woollacott and Shumway-Cook, 2002; Dubost et al., 2006) and subsequently increase the magnitude of gait-cognition associations (Killane et al., 2014). Previous research has documented such links between increased cognitive demands and corresponding impacts on age-graded impairments in physical function; the impact is greatest for older adults due to age-related increases in the demands of physical functions (less automatic and more effortful) for diminishing cognitive resources (Lövdén et al., 2008). Such explanations are consistent with our findings.

In future research, it will be important to directly explore potential mechanisms underlying why better gait function is protective against diminished cognitive decline. One promising avenue of study could involve cognitive reserve—a concept employed to help explain the vast individual differences in susceptibility to normative and pathological age-related cognitive decline (Stern, 2012). Recent research has explored the critical question as to why some individuals are more cognitively resilient than others, despite the presence of underlying brain changes. Although cognitive reserve is often indexed using a single indicator (years of education), multiple-indicator methods have also been employed (Opdebeeck et al., 2016). In one recent study by Grotz et al. (2017), cognitive reserve was operationalized as a multifaceted construct weighted by key indicators from across the lifespan including years of education, last occupation, and current participation in leisure activities. This weighted index of cognitive reserve more fully attenuated the age-cognition association, relative to education alone; based on this pattern, the weighted index was deemed to be the better proxy of cognitive reserve. Notably, Grotz et al. (2017) suggested that a negative load index, comprised of risk indicators such as chronic health conditions or body mass index, could further identify factors that negatively influence levels of cognitive reserve. Of direct relevance to the present study, diminished gait function may represent one such risk indicator with implications for reserve. In future studies, identifying key underlying indicators of cognitive reserve will be invaluable for identifying those who may be protected against various conditions from MCI (Franzmeier et al., 2016) to depression (Freret et al., 2015) with increasing age.

Among the study limitations, selective longitudinal survival likely influenced the results. Simple comparisons showed that the survivors reported slightly more years of education and better absolute self-reported health at baseline relative to their non-surviving cohort members (although levels were high for both groups). Further, despite the possible survival-related advantages, significant gait-cognition associations were observed for all cognitive outcomes, including for measures of incidental recall and semantic memory that are typically resistant to age-related influences. On balance, the observed patterns likely underestimate the true magnitude of associations between gait and cognitive decline. The sample size for the concurrent gait predictors and associated analytic constraints represent additional study limitations. Given the recent addition of the GAITRite assessment to the VLS protocol, our analyses were restricted to the examination of concurrent gait differences in relation to retrospective (prior) cognitive decline across as many as 25 years. We acknowledge the retrospective limitation of our design, but note that evidence of decline from a prior level of functioning represents important clinical detail, especially among higher functioning individuals, as well as the successful use of such designs in previous research (MacDonald et al., 2004). Despite the modest sample size for gait, the large number of assessments (*n* = 6–8) increased the statistical power to detect cognitive change (Rast and Hofer, 2014), and helped to offset this limitation.

In sum, findings from the present study represent a conservative first step for studying prospective gait-cognitive change relations. Contrasting prediction patterns for timed walk vs. normalized velocity from the first two research objectives extends recent findings underscoring the importance of objective gait assessment for use in *clinical* settings (Youdas et al., 2006) to *research* contexts as well, with important implications for reliable gait measurement and prospective identification of those at risk of various age-related outcomes. Findings from the second research objective extend gait-cognition associations longitudinally, thus addressing concerns that previously reported cross-sectional links are a spurious byproduct of between-subject age confounds. Patterns from the third research objective exploring the impact of dual-tasking on the moderating influence of gait on long-term cognitive change are consistent with conclusions drawn from cross-sectional studies. Dual tasking augments the moderating influence of gait on cognitive change, a finding consistent with greater attentional demands for maintaining gait and balance with increasing age (Lindenberger et al., 2000; Lövdén et al., 2008). Walking itself places ever-increasing demands on available cognitive processes; age-related constraints on such cognitive resources (particularly attentional processes) result in resource competition impacting motor control and coordination for even basic assessments of physical function (Woollacott and Shumway-Cook, 2002). Further, the increasing importance of gait variability as a predictor of cognitive change under dual-tasking conditions is consistent with recent findings that document increases in age-cognitive variability associations for an interference (but not control) condition of an executively demanding task, with the degree of attenuation of the age-cognitive variability association most pronounced after partialing estimates of dopamine binding for select regions in the cingulo-fronto-parietal (dorsal attention) network (MacDonald et al., 2012). Collectively, these findings underscore the importance of attentional processes as modulators of age-related increases in variability for the domains of physical function as well as cognition, and suggest that common mechanisms (e.g., age-related losses in dopamine binding potential) may underlie increases in both gait and cognitive variability (MacDonald et al., 2012; Rosso et al., 2013), as well as the predictive importance of variability indicators in their respective literatures. Looking to the future, research designs that incorporate various domains (cognition, gait, neural function) and indicators (mean, variability) will be best positioned for investigating the complex interrelations among aging, CNS, and physical and cognitive function, and their contributions to numerous age-related processes and outcomes—spanning successful aging to frailty and death (Amboni et al., 2003; Rosso et al., 2013).

## Ethics Statement

This research was conducted under full, active and continuous human ethics approval from prevailing Institutional Review Boards. The Human Research Ethics Board (HREB) of the University of Victoria approved this study. Written informed consent was obtained from all participants.

## Author Contributions

SWSM and RAD developed the theoretical research focus; SWSM analyzed the data and wrote the manuscript. SH, JAL, CAD, DWRH, PWHB, TVL, RC and RAD contributed to theoretical discussions and refinements, as well as provided detailed suggestions for revision of manuscript drafts.

## Funding

This research was supported by a grant from the National Institutes of Health/National Institute on Aging (R01 AG008235) to RAD, who also acknowledges support from the Canada Research Chairs program. SWSM was supported by grants from the National Institutes of Health/National Institute on Aging (R21 AG045575) and the Natural Sciences and Engineering Research Council of Canada (418676-2012).

## Conflict of Interest Statement

The authors declare that the research was conducted in the absence of any commercial or financial relationships that could be construed as a potential conflict of interest.
